# Nifedipine Treatment Reduces Resting Calcium Concentration, Oxidative and Apoptotic Gene Expression, and Improves Muscle Function in Dystrophic *mdx* Mice

**DOI:** 10.1371/journal.pone.0081222

**Published:** 2013-12-09

**Authors:** Francisco Altamirano, Denisse Valladares, Carlos Henríquez-Olguín, Mariana Casas, Jose R. López, Paul D. Allen, Enrique Jaimovich

**Affiliations:** 1 Centro de Estudios Moleculares de la Célula, Instituto de Ciencias Biomédicas, Facultad de Medicina, Universidad de Chile, Santiago, Chile; 2 Department of Molecular Biosciences, School of Veterinary Medicine, University of California Davis, Davis, California, United States of America; 3 Department of Anesthesiology, Perioperative and Pain Medicine, Brigham & Women’s Hospital, Harvard Medical School, Boston, Massachusetts, United States of America; 4 Programa de Fisiología y Biofísica, Instituto de Ciencias Biomédicas, Facultad de Medicina, Universidad de Chile, Santiago, Chile; University of Minnesota, United States of America

## Abstract

Duchenne Muscular Dystrophy (DMD) is a recessive X-linked genetic disease, caused by mutations in the gene encoding dystrophin. DMD is characterized in humans and in *mdx* mice by a severe and progressive destruction of muscle fibers, inflammation, oxidative/nitrosative stress, and cell death. In *mdx* muscle fibers, we have shown that basal ATP release is increased and that extracellular ATP stimulation is pro-apoptotic. In normal fibers, depolarization-induced ATP release is blocked by nifedipine, leading us to study the potential therapeutic effect of nifedipine in *mdx* muscles and its relation with extracellular ATP signaling. Acute exposure to nifedipine (10 µM) decreased [Ca^2+^]_r_, NF-κB activity and iNOS expression in *mdx* myotubes. In addition, 6-week-old *mdx* mice were treated with daily intraperitoneal injections of nifedipine, 1 mg/Kg for 1 week. This treatment lowered the [Ca^2+^]_r_ measured *in vivo* in the *mdx vastus lateralis.* We demonstrated that extracellular ATP levels were higher in adult *mdx flexor digitorum brevis* (FDB) fibers and can be significantly reduced after 1 week of treatment with nifedipine. Interestingly, acute treatment of *mdx* FDB fibers with apyrase, an enzyme that completely degrades extracellular ATP to AMP, reduced [Ca^2+^]_r_ to a similar extent as was seen in FDB fibers after 1-week of nifedipine treatment. Moreover, we demonstrated that nifedipine treatment reduced mRNA levels of pro-oxidative/nitrosative (iNOS and gp91^phox^/p47^phox^ NOX2 subunits) and pro-apoptotic (Bax) genes in *mdx* diaphragm muscles and lowered serum creatine kinase (CK) levels. In addition, nifedipine treatment increased muscle strength assessed by the inverted grip-hanging test and exercise tolerance measured with forced swimming test in *mdx* mice. We hypothesize that nifedipine reduces basal ATP release, thereby decreasing purinergic receptor activation, which in turn reduces [Ca^2+^]_r_ in *mdx* skeletal muscle cells. The results in this work open new perspectives towards possible targets for pharmacological approaches to treat DMD.

## Introduction

Duchenne muscular dystrophy (DMD) is a severe neuromuscular disorder characterized by the absence of the dystrophin protein and is the most common form of muscular dystrophy with a frequency of 1 in 3500 male births [1]. DMD is caused by a recessive X-linked mutation in dystrophin gene located to Xp21 [2]. Usually patients lose the ability to walk between 6–12 years old due to severe muscle damage and contractures that results in wheelchair dependence [1]. Since DMD is a progressive disease, most patients die in their twenties due either to respiratory failure or cardiac dysfunction [3].

Resting intracellular calcium concentration ([Ca^2+^]_r_) is elevated in myotubes from *mdx* mice, a murine model for DMD [4]. This increased [Ca^2+^]_r_ depends on sarcolemmal Ca^2+^ entry, as well as Ca^2+^ leak from sarcoplasmatic reticulum (SR), through type 1 ryanodine receptors (RyR1) and inositol tri-phosphate receptors (IP_3_R). In turn, elevated [Ca^2+^]_r_ modulates NF-κB activation, leading to an up-regulation of inducible nitric oxide synthase (iNOS) in dystrophic myotubes [4].

The L-type voltage gated Ca^2+^ channels (Dihydropyridine receptors, DHPRs) are also implicated in DMD pathology [5]–[7]. Their roles as voltage sensors for both excitation-contraction and excitation-transcription coupling, and as their role as L-type Ca^2+^ channels in skeletal muscle cells, make them key regulators of intracellular [Ca^2+^] [8]–[10]. The DHPR is also an important modulator of ATP release through Pannexin1 channels in skeletal muscle fibers [9], [10]. ATP release elicited by electrical stimuli is blocked with the DHPR antagonist nifedipine as well as with the agonist (−)S-BayK 8644 in FDB fibers [10], suggesting that DHPR could directly control ATP release and that this event is independent of L-type current. An interaction between dystrophin and DHPR has been proposed in transverse tubular system of skeletal muscle fibers [6], [11], suggesting that there is DHPR dysregulation in dystrophic skeletal muscle cells. More recently, we showed that basal levels of ATP release are importantly increased in *mdx* muscle fibers [12]. In dystrophic fibers, extracellular ATP stimulation was pro-apoptotic, inducing the transcription of Bax, BIM and PUMA and increasing the levels of activated Bax and cytosolic cytochrome C [12]. These data suggest the potential for involvement of the ATP pathway in the activation of mechanisms related with cell death in muscular dystrophy, opening new perspectives towards possible targets for pharmacological therapies.

A double-blinded controlled clinical trial with nifedipine was carried out in 1987, and showed that there was no significant beneficial effect of nifedipine treatment [13]. In this study, patient selection was restricted to semiology and classical human genetics, since dystrophin was not cloned until 1987 [2]. DMD has been considered stereotyped in its clinical presentation, evolution and severity [14]. However, an inverse correlation between severity of disease and residual amount of dystrophin (related to different mutations) has been found [15]. More recently, a retrospective, single institution, long-term (>10 years) study was done in 75 DMD patients with a complete lack of dystrophin (determined by western blot) and genotyping, showed that DMD can be divided into 4 sub-phenotypes with different cognitive and motor outcomes [16], showing the complexity and heterogeneity of DMD.

Based on our previous results showing that extracellular ATP levels are elevated in *mdx* fibers and that ATP stimulation is pro-apoptotic, here we revisited the potential therapeutic effect of this drug in the *mdx* mouse model. In the present study, we found that acute treatment of *mdx* myotubes with nifedipine reduced [Ca^2+^]_r_, NF-κB activity and iNOS expression. Likewise, intraperitoneal injections of nifedipine for 1 week, reduced *in vivo* [Ca^2+^]_r_ in *vastus lateralis* muscles, decreased serum creatine kinase levels and increased *in vivo* muscle strength in *mdx* mice. In FDB muscle fibers isolated from the same nifedipine treated *mdx* mice, basal ATP release was reduced and the gene expression of pro-oxidative and pro-apoptotic genes in diaphragm (the most seriously affected muscle) were down-regulated. The present findings demonstrate that nifedipine treatment can effectively modify the observed changes associated with muscle pathology in *mdx* mice and opens a new pharmacologic approach for treatment of patients with DMD.

## Materials and Methods

### Myotube Cultures

Primary myoblasts were isolated from 5–6 week old male wild type C57BL/6 and *mdx* mice and myotubes were differentiated as described previously [4], [17].

### Adult FDB Fiber Isolation

*Flexor digitorum brevis* (FDB) muscles were dissected from 5–6 week old male mice and intact muscle fibers were obtained by enzymatic digestion of the whole muscle with collagenase type 4 (Worthington, Lakewood, NJ) for 90 min at 37°C followed by mechanical dissociation with fire polished Pasteur pipettes. Isolated fibers were seeded in matrigel-coated dishes in DMEM supplemented with 10% horse serum and used for experimentation 20–24 hours after isolation.

### Drug Treatment Protocol

Male 5–6 week-old *wt* and *mdx* mice were injected daily for 1 week with either nifedipine solution (1 mg/Kg body weight) or saline intraperitoneally. Nifedipine (Sigma-Aldrich) solution was prepared in dark at 17 mg/mL in absolute ethanol and then was diluted in sterile saline solution (0.9% NaCl) at 0.2 mg/mL for injections.

### Determination of [Ca^2+^]_r_

Double-barreled Ca^2+^-selective microelectrodes were prepared and calibrated as previously described [4]. Pulled microelectrodes were backfilled with the neutral carrier ETH 129 (Fluka-Sigma-Aldrich) and then with pCa7 solution. Only those electrodes with a linear relationship between pCa3 and pCa7 (Nernstian response, 29.5 mV and 30 mV per pCa unit at 23°C and 37°C, respectively) were used experimentally.

Myotubes or muscle fibers were impaled with the double-barreled Ca^2+^ selective microelectrodes and potentials were recorded via high impedance amplifier (WPI Duo-773) [18]. [Ca^2+^]_r_ measurements in myotubes were made with or without nifedipine (10 µM) in Krebs-Ringer solution (in mM: 140 NaCl, 5 KCl, 2.5 CaCl_2_, 1 MgSO_4_, 5 glucose, and 10 Hepes/Tris, pH 7.4) at 23°C, as previously described [4].

The measurements of [Ca^2+^]_r in muscle fibers_ were done *in vivo* in the *vastus lateralis*. Mice were anesthetized with ketamine 100 mg/Kg and xylazine 5 mg/Kg and kept euthermic with a feedback-heating pad (ATC-1000 Temperature controller, WPI, Sarasota, FL). A small incision was made in the skin in the anterior part of the left leg, the *vastus lateralis* muscle was identified and fascia was partially removed. The superficial fibers were exposed and locally perfused with Krebs Ringer solution.

Measurements of [Ca^2+^]_r_ in isolated adult FDB fibers were done after fibers were incubated in Krebs Ringer solution with or without apyrase 2 U/mL (grade VII from potato, Sigma-Aldrich) for 10 min at room temperature.

### NF-κB Luciferase Reporter Activity

Both *wt* and *mdx* myoblasts were transduced with a lentivirus containing 5 tandem NF-κB binding site repeats cloned upstream of a luciferase reporter gene and populations that stably expressed the transgene were selected using G418, as described previously [4]. Myoblasts stably expressing this reporter were completely normal and differentiated into myotubes after 3–4 days similar to untransduced cells. Myotubes were treated with or without nifedipine for 6 h in differentiation media. Luciferase activity was determined using a dual-luciferase reporter assay system (Promega) according to the manufacturer’s instructions, and light detection was carried out in a Berthold F12 luminometer. Results were normalized with total protein and the relations “luciferase activity/mg protein” were shown.

### mRNA Quantitation

Total RNA was isolated from myotubes or diaphragm muscles from both the nifedipine- or saline-treated groups with TRIzol® reagent (Invitrogen) according to the manufacturer’s protocol. cDNA was prepared by reverse transcription (RT) reaction of 1 µg of total RNA using random primers. Real time PCR was performed as previously described [4] using the following primers:

*bax 5′-* GCTGACATGTTTGCTGATGG-3′ and 5′-GATCAGCTCGGGCACTTTAG-3′ *bim* 5′-CGACAGTCTCAGGAGGAACC-3′ and 5′-CATTTGCAAACACCCTCCTT-3′ *gp91^phox^* 5′-TCACATCCTCTACCAAAACC-3′ and 5′-CCTTTATTTTTCCCCATTCT-3′ *p47^phox^* 5′-AGAACAGAGTCATCCCACAC-3′ and 5′-GCTACGTTATTCTTGCCATC-3′ *iNOS* 5′-CAGCTCAAGAGCCAGAAACG-3′ and 5′-TTACTCAGTGCCAGAAGCTG-3′ *gapdh* 5′-CTCATGACCACAGTCCATGC-3′ and 5′-TTCAGCTCTGGGATGACCTT-3′ *18S rRNA* 5′-GGGCCCGAAGCGTTTACTTT-3′ and 5′-TTGCGCCGGTCCAAGAATTT-3′.

### ATP Detection Using a Luciferin/Luciferase Assay

FDB fibers from nifedipine- and saline-treated mice were prepared as described above. Because media replacement causes a mechanical stimulus that itself induces ATP release [19], [20], we measured ATP release for up to 9 min beginning 30 min after media change. ATP concentrations were measured with the CellTiter-Glo® Luminescent Cell Viability Assay (Promega, Madison, WI, USA) as reported [9]. Data were calculated as pmol extracellular ATP/µg total RNA. Normalization by total RNA instead of total protein was chosen because fibers were seeded on a Matrigel-coated dish (containing a large amount of protein), which may affect the protein determination associated only to fibers.

### Serum Creatine Kinase Determinations

Blood samples were obtained by cardiac puncture in anesthetized mice. Blood was collected in a sterile test tube, allowed to clot on ice for 30 min and then centrifuged at 3000 rpm for 10 minutes. Creatine kinase (CK) levels were determined using the UV-kinetic method (Teco Diagnostics) according to the manufacturer instructions. ΔAbs/min were used to calculate CK enzymatic activity and the results were expressed as International Kilo Units per liter (KUI/L).

### Inverted Grid-hanging Test

Muscle strength of mouse limbs was tested with an inverted grid-hanging test [21]. Mice were placed individually on the center of a 21×21 cm wire grid (wire width ≈0.1 cm and spacing 0.5 cm), mounted 35 cm above a table. After gently inverting the grid, the mouse hanging time was recorded (grip latency). This procedure was repeated three times and the average hanging time values were calculated for each mouse.

### Forced Swimming Test

A 1-liter beaker (11 cm diameter and 15 cm height) filled with water (23°C) was used as swimming pool to assess the exercise tolerance of saline- and nifedipine-treated mice [22]. First, a weight (10% of their body weight) was attached to the tail of a mouse, which was then gently placed in the water, and the time at which the mouse was unable to maintain complete buoyancy was recorded. At this time the mice were immediately removed from the swimming pool, dried gently with paper towels and returned to their cages.

### Statistical Analysis

Data of *n* experiments were expressed as mean ± S.E.M. The significance of difference among treatments was evaluated using a *two-tailed t* test for unpaired data or ANOVA- followed by *Tukey’s t-test*. A *P* value <0.05 was considered statistically significant.

### Ethics Approval

All procedures for animal experimentation were done in accordance with guidelines approved by the Bioethical Committee at the Facultad de Medicina, Universidad de Chile and the IACUCs at Harvard Medical School and University of California at Davis.

## Results

### Nifedipine Reduces [Ca^2+^]_r_ in Dystrophic *mdx* Myotubes

**Figure 1:**
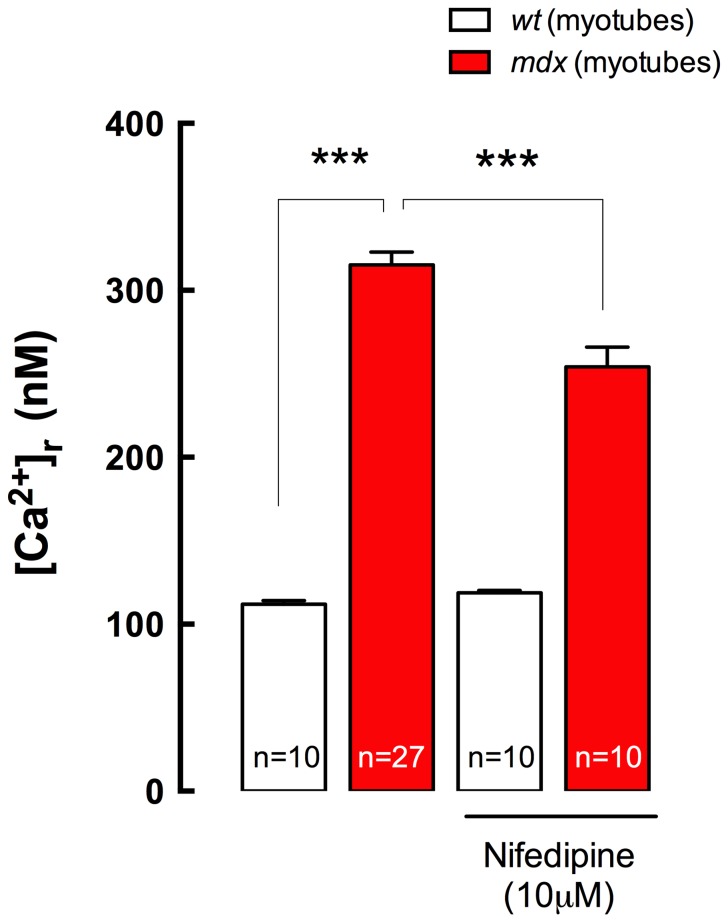
Nifedipine incubation reduces [Ca^2+^]_r_ in *mdx* myotubes. Myotubes were incubated with 10 µM Nifedipine for 10 min at room temperature in Krebs Ringer solution and [Ca^2+^]_r_ was measured using double-barreled Ca^2+^ selective microelectrodes. Data are expressed as mean ± S.E.M. *Wt* (n = 10), *mdx* (n = 27), *Wt*+Nife (n = 10) and *mdx+*Nife (n = 10). ***P<0.001, ANOVA-*Tukey’s.*

Myotubes were incubated in Krebs Ringer solution with or without nifedipine (10 µM) for 10 min and [Ca^2+^]_r_ was measured with Ca^2+^ selective microelectrodes, in both *wt* and *mdx* myotubes. [Ca^2+^]_r_ observed in *mdx* myotubes was significantly higher compared to *wt* myotubes (315±8 *vs* 112±2 nM *P*<0.001) (Figure 1). There was a significant reduction in the [Ca^2+^]_r_ in nifedipine-treated *mdx* myotubes compared with untreated *mdx* myotubes (254±12, *P<*0.001). Nifedipine treatment did not modify [Ca^2+^]_r_ in *wt* myotubes (119±1 nM, *P>*0.05).

### NF-κB Activity and iNOS Expression were Reduced After Nifedipine Incubation

**Figure 2:**
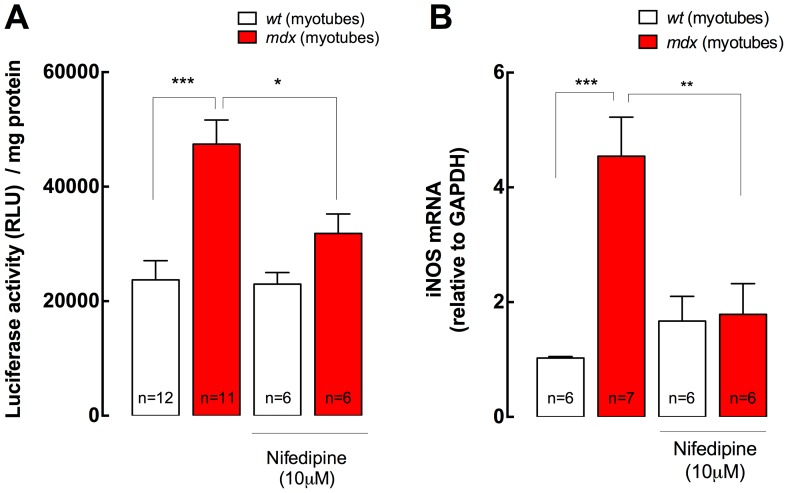
NF-κB activity and iNOS expression in both *wt* and *mdx* myotubes. **A.** NF-κB activity was studied with a luciferase reporter. Nifedipine treatment (10 µM for 6 h) reduced NF-κB activity in *mdx*, without any significant effect in *wt* myotubes (n = 6–12). **B.** mRNA levels of iNOS were determined by real time PCR after 6 h of nifedipine treatment (10 µM) (n = 6–7). Data are expressed as mean ± S.E.M., *P<0.05, **P<0.01, ***P<0.001, ANOVA-*Tukey’s.*

We previously reported that the increases in NF-κB activity and iNOS expression are modulated by [Ca^2+^]_r_ in *mdx* myotubes [4]. To study the effect of nifedipine treatment in NF-κB activity and iNOS expression, we used a NF-κB luciferase reporter and real time PCR, respectively (see *Materials and Methods*). After incubation with nifedipine (10 µM, 6 h) NF-κB activity was reduced by 33% (*P<*0.05) and iNOS mRNA levels were diminished by 61% (*P<*0.01) in *mdx* myotubes, without any significant effect in *wt* myotubes (*P>*0.05) (Figure 2).

### Nifedipine Decreases the [Ca^2+^]_r_ *in vivo* in *mdx* Muscles

**Figure 3:**
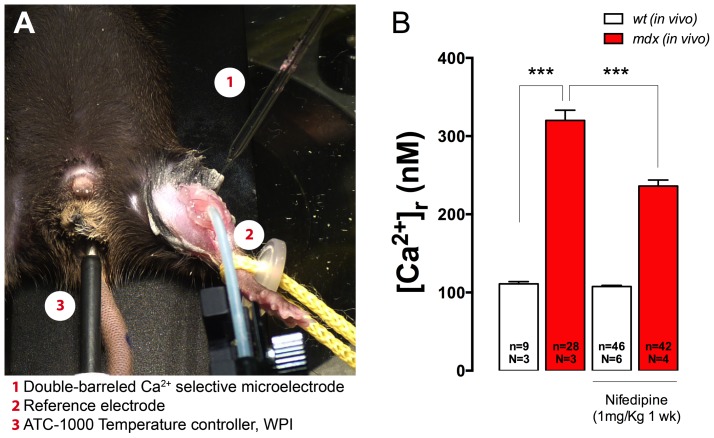
Nifedipine treatment reduces muscle [Ca^2+^]_r_ *in vivo* in *mdx* mice. Mice were treated with daily intraperitoneal injections of nifedipine 1/Kg for 1-week or saline. **A.** Experimental setup to measure [Ca^2+^]_r_ *in vivo* with Ca^2+^ selective microelectrodes in *vastus lateralis* muscle. **B.** Averaged data from [Ca^2+^]_r_ determinations with or without nifedipine treatment. Data are expressed as mean ± S.E.M. from *n* fibers in *N* mice, ***P<0.001, ANOVA-*Tukey’s.*

To establish if nifedipine can reduce [Ca^2+^]_r_ *in vivo*, both *wt* and *mdx* mice were injected intraperitoneally daily for 1 week with either 1 mg/Kg nifedipine solution or saline. [Ca^2+^]_r_ measured *in vivo* in the superficial fibers of *vastus lateralis* muscles was significantly higher in *mdx* muscles compared with *wt* muscles in the saline treated group (Figure 3, 320±13 *vs* 111±3 nM, *P<*0.001). Nifedipine treatment was able to significantly reduce [Ca^2+^]_r_ in *mdx* mice (236±8 nM, *P<*0.001)_,_ but had no effect in *wt* muscles (108±1 nM, *P>*0.05).

### Nifedipine Treatment Reduces ATP Release in Adult Fibers

**Figure 4:**
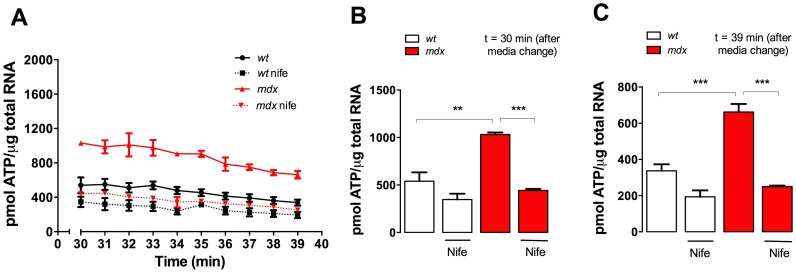
Extracellular ATP concentration in FDB fibers isolated from either nifedipine- or saline-treated *wt* and *mdx* mice. **A.** Time course of extracellular ATP levels after media change. ATP concentration was measured with CellTiter-Glo® Luminescent Cell Viability Assay. Average extracellular ATP at 30 min (**B**) and at 39 min (**C**) after media change are shown in the figure. Data are expressed as mean ± S.E.M. FDB fibers were cultured from *n* = mice are indicated in the figure. **P<0.01, ***P<0.001, ANOVA-*Tukey’s*.

Recently, we reported that depolarization-induced ATP release is modulated by DHPR activity and can be blocked in skeletal muscle cells by nifedipine [10]. Moreover, extracellular ATP levels are higher at resting conditions in *mdx* skeletal muscle fibers [12]. It is very well established that extracellular ATP can signal through purinergic receptors and modulate the intracellular Ca^2+^ concentration [23]. In order to establish a correlation between extracellular ATP and the alterations in the [Ca^2+^]_r_, we measured ATP release in isolated FDB fibers from both *wt* and *mdx* mice after 1-week of nifedipine or saline treatment. Because media replacement causes a mechanical stimulus that itself induces ATP release [19], [20], we measured ATP release after 30 min of media change and then up to 9 min. ATP release from *mdx* fibers was higher compared to *wt* fibers at every studied time point (Figure 4A). Nifedipine treatment reduced ATP release in both *wt* and *mdx* fibers, with a more pronounced effect in *mdx* fibers. At t = 30 min after media change average extracellular ATP in fibers from saline-treated *mdx* mice was higher than in saline-treated *wt* fibers (1031±22 *vs* 540±93 pmol ATP/µg RNA), *P<*0.01) (Figure 4B). ATP release was significantly decreased in fibers isolated from nifedipine-treated *mdx* (442±18 pmol ATP/µg RNA, *P<*0.001) compared to untreated *mdx* fibers. Similar results were observed after 9 min (Figure 4C), showing that extracellular ATP levels were higher in *mdx* fibers but can be diminished near to *wt* levels after 1-week of nifedipine treatment.

### Degradation of Extracellular ATP Reduces [Ca^2+^]_r_ in *mdx* Adult Fibers

**Figure 5:**
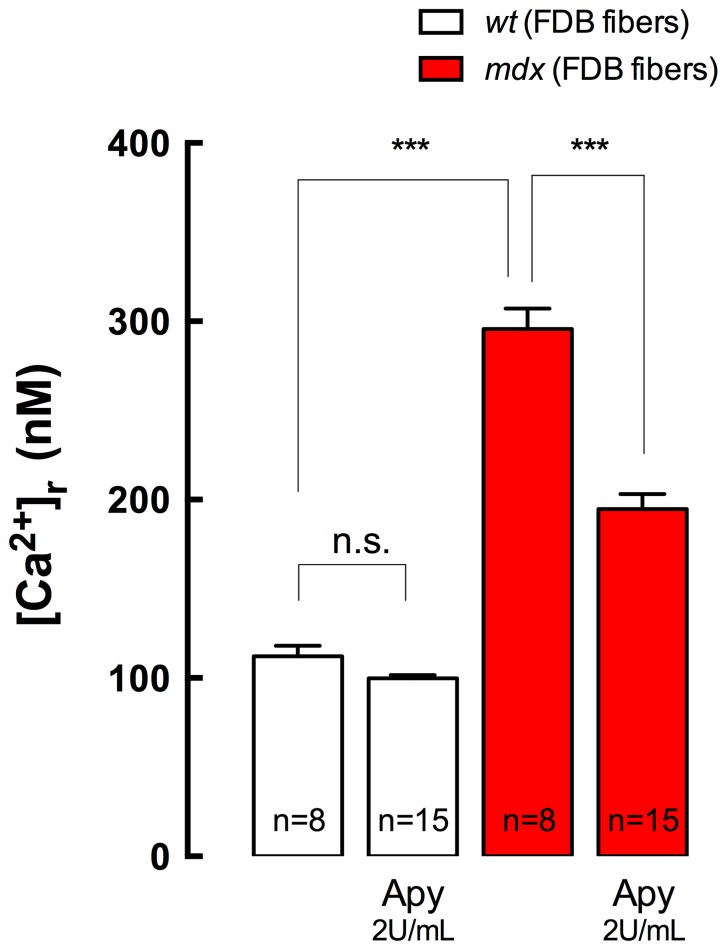
Apyrase treatment reduces [Ca^2+^]_r_ in FDB adult fibers. Adult fibers were isolated from *wt* and *mdx* mice and incubated in Krebs Ringer solution with or without apyrase (2 U/mL) for 10 min at room temperature. [Ca^2+^]_r_ was determined by double-barreled Ca^2+^ selective microelectrodes. Data are expressed as mean ± S.E.M. from *n* fibers (indicated in the figure) from three different cultures. n.s, no significant difference, ***P<0.001, ANOVA-*Tukey’s*.

In order to determine the participation of extracellular ATP on [Ca^2+^]_r_ in adult *mdx* fibers, we treated isolated FDB fibers with apyrase (2 U/mL) in Krebs Ringer solution for 10 min. Apyrase is an enzyme that rapidly metabolizes extracellular ATP to AMP [9]. Apyrase treatment significantly reduced [Ca^2+^]_r_ from 296±11 to 195±8 nM (*P*<0.001) in *mdx* fibers, without any significant effect in *wt* fibers (112±6 to 100±2 nM, *P*>0.05) (Figure 5).

### NADPH Oxidase Subunits and iNOS Expression in *mdx* Mice

**Figure 6:**
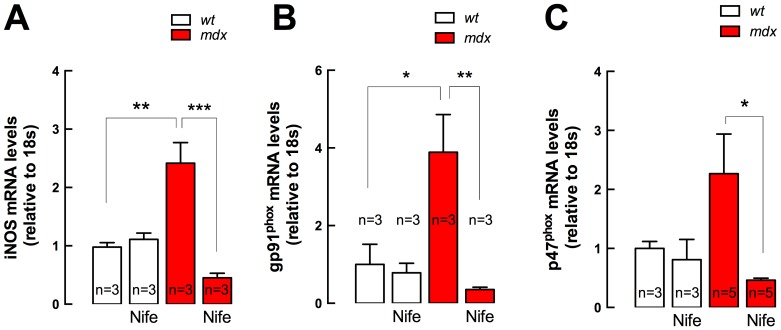
iNOS and NADPH oxidase subunits gene expression in diaphragm muscles. Diaphragms were dissected from nifedipine- and saline-treated mice and mRNA levels were assessed by real time PCR. **A.** iNOS, **B.**, gp91^phox^ and **C.** p47^phox^ expression. Data are expressed as mean ± S.E.M. Diaphragms were obtained from *n* = mice as indicated in the figure. *P<0.05, **P<0.01, ***P<0.001, ANOVA-*Tukey’s*.

Dystrophic muscles are characterized by an increase in pro-oxidative gene expression, such as iNOS and NADPH oxidase subunits (gp91^phox^, p67^phox^ and rac1) [4], [24], [25]. Because the diaphragm has been described as the most severely affected muscle in the *mdx* mouse [26] it was used to determine the antioxidant effects of nifedipine treatment. For these studies we determined mRNA levels of iNOS and the NADPH oxidase subunits gp91^phox^ and p47^phox^, from whole diaphragm lysates using real time PCR. We found that iNOS mRNA levels were 2.4-fold and gp91^phox^ were 3.9-fold higher in *mdx* diaphragms compared to *wt*, with no significant difference in the expression of p47^phox^ between the two groups (Figure 6). Nifedipine treatment reduced the mRNA levels of iNOS, gp91^phox^ and p47^phox^ in *mdx* diaphragms by 86% (*P<*0.001), 91% (*P<*0.01) and 80% (*P<*0.05), respectively, with no significant effect in *wt* muscles.

### Nifedipine Treatment Normalizes Pro-apoptotic Genes Expression in *mdx* Mice

**Figure 7:**
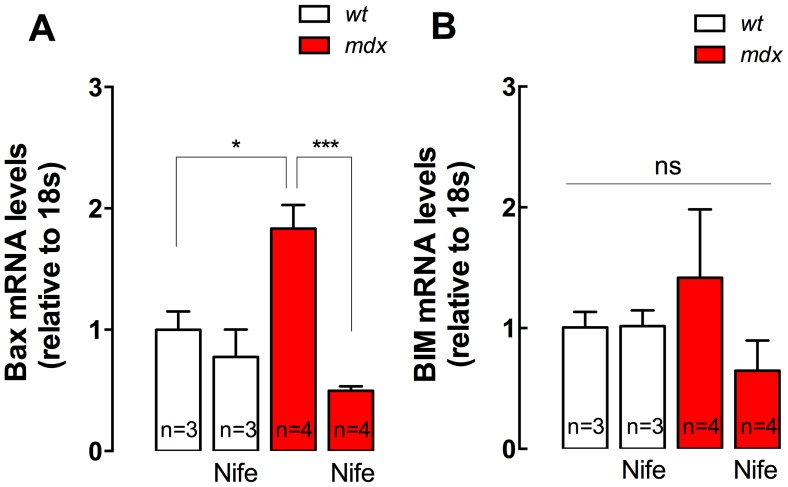
Bax and BIM gene expression in diaphragm muscles. Diaphragms were dissected from nifedipine- and saline-treated mice and mRNA levels were assessed by real time PCR. **A.** Bax, **B.** BIM mRNA levels. Data are expressed as mean ± S.E.M. Diaphragms were obtained from *n* = mice as indicated in the figure, *P<0.05, ***P<0.001, ANOVA-*Tukey’s*.

Necrosis is probably one of the major contributors to DMD pathology [27]. However, there is some evidence that suggests that apoptotic pathways could also be important [28], [29]. To determine if nifedipine can modify pro-apoptotic gene expression, we determined mRNA levels of both Bax and BIM in diaphragm muscles using real-time PCR. Bax mRNA levels were higher in *mdx* compared with *wt* mice (2.0-fold, *P<*0.05) (Figure 7) and nifedipine administration significantly diminished mRNA levels of Bax in *mdx* diaphragm by 73% (*P<*0.001) with no significant effect in *wt* diaphragm. We did not find any differences in BIM mRNA levels between any of the studied groups.

### Nifedipine Treatment Diminishes Serum CK Levels and Improves Muscle Strength in *mdx* Mice

**Figure 8:**
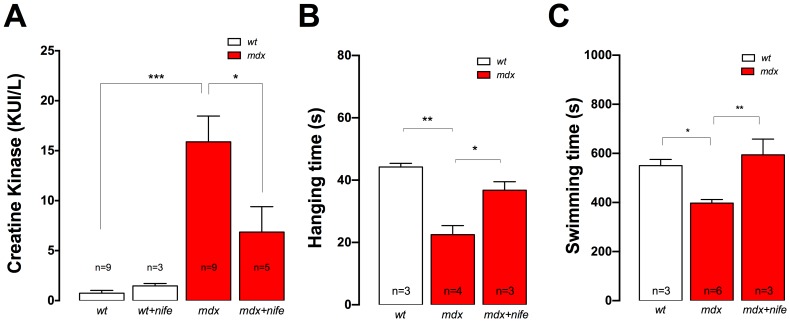
Nifedipine treatment reduced serum CK and increases muscle function in *mdx* mice. **A.** Blood samples were collected by cardiac puncture under anesthesia from both nifedipine- or saline-treated mice. CK activities were determined by the UV kinetic method. **B.** Averaged hanging time obtained in the inverted grid-hanging test in saline- and nifedipine treated mice. **C.** Averaged swimming time obtained in the forced swimming test in nifedipine- or saline- treated mdx mice. Data are expressed as mean ± S.E.M. *n* = mice is indicated in the figure, *P<0.05, **P<0.01, ***P<0.001 ANOVA-*Tukey’s*.

To elucidate whether nifedipine treatment could reduce muscle damage, we measured serum CK levels in both *wt* and *mdx* mice after 1 week of either saline or nifedipine treatment. CK levels were significantly higher in saline treated *mdx* mice relative to saline treated *wt* mice (15.9±2.6 *vs* 0.7±0.3 KUI/L, *P<*0.001) (Figure 8A). Nifedipine reduced CK levels in *mdx* mice (6.8±2.5 KUI/L, *P<*0.05), without any significant effect in *wt* mice (1.5±0.2 KUI/L, *P>*0.05).

In order to determine whether nifedipine treatment might increase muscle strength we used the inverted grid-hanging test and the forced swimming test. Mice were treated daily with nifedipine (1 mg/Kg) or saline for 1 week, and the functional test was performed one day after cessation of treatment. Saline treated *mdx* mice had a significantly shorter hanging time compared to *wt* mice (23±3 s vs 44±1 s, *P*<0.01 Figure 8B). Nifedipine treatment increased the mdx hanging time by 61% (37±3 s, *P*<0.05 compared to saline-treated mice (Figure 8B).

Similar results were observed using the forced swimming test. Under this experimental condition, *wt* swimming time was significantly higher compared to saline-treated *mdx* mice (550±25 s *vs* 398±15 s, *P*<0.05, Figure 8C). In this case nifedipine treatment for 1 week normalized the swimming time in *mdx* mice increasing it to 594±64 s (*P*<0.01 compared to saline-treated mice, Figure 8C).

## Discussion

Our data show that [Ca^2+^]_r_ was elevated in *mdx* myotubes and to a similar extent in adult skeletal muscle fibers from *mdx* mice. Acute exposure of *mdx* myotubes to nifedipine decreased [Ca^2+^]_r_, NF-κB activity and iNOS expression. Likewise, in *mdx* mice, nifedipine treatment for 1-week lowered *in vivo* [Ca^2+^]_r_ in the *vastus lateralis,* reduced ATP release in FDB fibers, diminished mRNA levels of pro-oxidative/apoptotic genes in the diaphragm, lowered serum CK levels and improved muscle function as assessed by both inverted grid-hanging test and forced swimming test.

Although still controversial, it is generally accepted that dystrophic skeletal muscle cells have elevated [Ca^2+^]_r_ [4], [30]–[32]. Our data support these findings showing that [Ca^2+^]_r_ was elevated in *mdx* myotubes and to a similar extent in skeletal muscles from *mdx* mice, measured either in isolated FDB fibers or determined *in vivo,* in anesthetized mice. Moreover, we showed that either acute nifedipine treatment of myotubes or chronic nifedipine treatment of mice for 1-week reduced [Ca^2+^]_r_. Nifedipine is known as specific inhibitor of the DHPR [33]. In addition to its role as an L-type Ca^2+^ channel the DHPR operates as voltage sensor for both excitation-contraction and excitation-transcription coupling in adult muscle fibers [8]–[10]. Dystrophic *mdx* skeletal muscle cells have a dysregulated excitation-contraction coupling as evidenced by reduced calcium transients evoked by single action potentials compared with *wt* fibers [34], [35]. This has been used to explain the muscle weakness observed in DMD patients. On the other hand, there is a controversy related to whether there are alterations of L-type Ca^2+^ currents in *mdx* muscles, with some studies showing normal maximum currents [5] while others show decreased maximum current [6], [7]. No differences have been found in charge movement between normal and dystrophic skeletal muscle fibers [5], [36]. In addition, a leftward-shift of the voltage dependence of L-type Ca^2+^ current has been described [5].

Our data showed that extracellular ATP levels were higher in *mdx* fibers compared to *wt* fibers. Chronic nifedipine treatment reduces ATP release in adult *mdx* FDB fibers to a level similar to *wt* fibers. Moreover, acute enzymatic ablation of extracellular ATP-ADP with apyrase treatment reduces the [Ca^2+^]_r_ in *mdx* fibers from 296 nM to 195 nM, showing that elevated extracellular ATP is an important modulator of [Ca^2+^]_r_ in *mdx* skeletal muscle fibers. Extracellular ATP can signal through P_2_X (inotropic) and P_2_Y (metabotropic) purinergic receptors [23], [37], the latter pathway leading to Ca^2+^ release through inositol tri-phosphate receptors (IP_3_R). We previously reported that IP_3_ levels were higher in both human DMD and *md*x cell lines [38] and that either phospholipase C (U-73122) or IP_3_R (Xestospongin C) inhibition could significantly reduce [Ca^2+^]_r_ in *mdx* myotubes [4].

Pannexin-1 channels are involved in ATP release after depolarization in myotubes and adult muscle fibers [9], [10]. Based on co-immunoprecipitation and a proximity ligation assay [10], an interaction between DHPR and Pannexin-1 channels has been proposed. Recently, we have shown that the DHPR is an important modulator of ATP release, and that it is necessary for fast-to-slow phenotype transition in FDB adult muscle fibers stimulated at 20 Hz [10]. ATP release through pannexin-1 channels observed after 20 Hz electrical stimulation of adult muscle fibers is inhibited by nifedipine [10], suggesting that the DHPR could directly control ATP release via Pannexin-1 channels.

Due to a chronic state of fiber injury, dystrophic muscle would be expected to contain high levels of extracellular ATP. In addition, ATP release to the extracellular medium was shown to be elevated in muscle fibers isolated from *mdx* mice [12]. Several alterations have been found in ATP signaling in dystrophic skeletal muscle cells. In an immortalized myoblast cell line derived from *mdx* mouse, addition of exogenous ATP to the media induced a large increase in cytosolic Ca^2+^ concentration compared with its *wt* counterpart [39]. This increased susceptibility to ATP was associated with changes in expression and function of P_2_X channels and to pathogenic Ca^2+^ entry in dystrophic muscles. Furthermore, enhanced expression of the P_2_X_4_ and P_2_X_7_ receptors have been related to macrophage invasion in *mdx* mice [40].

Here we determined the expression of iNOS and NADPH oxidase subunits in diaphragm, because this muscle has been described as the most severely affected muscle in the *mdx* mouse [26]. Nifedipine reduced iNOS, gp91^phox^ and p47^phox^ mRNA levels in *mdx* diaphragm after 1-week of treatment. We previously demonstrated that iNOS expression was higher in *mdx* myotubes which we attributed to an up-regulated NF-κB activity mediated by the elevated [Ca^2+^]_r_ [4]. Nifedipine treatment reduced iNOS expression in diaphragm, likely due to the same mechanism.

Recently, it has been shown that dihydropyridines have anti-inflammatory and anti-oxidant effects. Nifedipine caused concentration-dependent inhibitory effects on sarcolemmal lipid peroxidation *in vitro* [41]. Toma *et al*, 2011 suggest that amlodipine, a third generation dihydropyridine L-type calcium channel blocker, may improve endothelial dysfunction in diabetics through anti-oxidant and anti-inflammatory mechanisms. The authors stimulated human endothelial cells with irreversible glycated low-density lipoproteins (AGE-LDL), as an *in vitro* model mimicking the diabetic condition. Their results show that amlodipine reduced the expression of NADPH oxidase subunits (p22^phox^ and NOX4) and iNOS, and diminished oxidative/nitrosative stress. Moreover, amlodipine had anti-inflammatory effects reducing the activation of MCP-1, VCAM-1, p38 MAPK and NF-κB [42]. In another study, it has been shown that amlodipine (3 mg/kg/day) decrease the expression of NADPH subunits (p47^phox^ and rac1), NADPH activity and the expression of inflammatory factor in atherosclerotic lesions [43].

Necrosis is probably the major contributor to DMD pathology [27]. However, there is some evidence that suggests that apoptotic pathways could also be important [28], [29]. We observed that nifedipine reduced significantly Bax expression in *mdx* diaphragms, suggestive of a protective anti-apoptotic effect. Bax was abundantly expressed in the *mdx* masseter muscles compared to *wt* muscles at 3 weeks after birth [29]. We have demonstrated that exogenous ATP increases mRNA levels of several pro-apoptotic genes in skeletal muscle fibers, including Bax, BIM and PUMA. Moreover, ATP induced Bax activation and cytochrome C release in *mdx* fibers, which is related with apoptotic cell death [12].

A previous double-blind controlled clinical trial with nifedipine did not demonstrate a beneficial therapeutic response on mean muscle strength, joint contractures, time-functional tests, pulmonary function, or creatine kinase levels in 105 patients between 3–27 years of age at the end of the study who were presumed to have DMD [13]. Patients were given nifedipine 0.75 mg/kg/day in three divided oral doses for 6 months and then 1.5–2 mg/Kg/day for the final 12 months of the study [13]. A total of 97 patients completed the trial. The power of the study to detect a difference between groups was calculated using the decrease of average muscle strength over time as the primary outcome measure. Average muscle strength score were calculated with a modified manual testing scale (MMT) [14] and the study had a power greater that 0.99 to detect a slowing of the illness to 25% of its original progression. The diagnostic criteria were based on semiology and classical human genetics, however three patients classified as Becker dystrophy were included in the study. Moreover the one patient aged 27 years old was considered to be unusual for DMD [44]. The data presented showed that the average muscle strength score as slightly improved without any significant difference. The trial used an unpaired analysis and it may have been more appropriate to use a paired analysis so that before and after changes in individuals could be compared [44].

As we mentioned before, there is an inverse correlation between severity of disease and dystrophin expression [15] and even patients with a complete lack of dystrophin DMD can be divided into 4 sub-phenotypes with different cognitive and motor outcomes [16]. This shows the high variability in the natural history of DMD. Phenotypic variations have shown to compromise results of clinical trials [45]. Desguerre *et al*, suggest that trials, which are in danger of being inconclusive due to lack of precise knowledge of DMD’s natural history, would strongly benefit from accurate selection of clinically homogeneous patient subsets [16].

High CK levels observed in DMD patients are attributed to an enhanced membrane permeability and muscle damage [46]. In our study, nifedipine treatment significantly reduced CK levels in *mdx* mice after 1-week, but did not normalize it. These data are supported by a study using verapamil, another L-type calcium channel blocker, which showed a significant reduction in the release of CK and LDH from diaphragm and gastrocnemius muscles [47]. In the previous nifedipine clinical trial, authors did not observe any significant difference in averaged CK levels after treatment, reporting that patients from nifedipine group had an average CK level of 2681 U/liter at the start and a value of 2045 U/liter at the end [13]. High levels of CK in DMD are well-known, and after a rise in infancy, they remain at high levels with an abrupt decrease at around 10 years old due to loss of muscle mass [48]. Thus averaged data from a broad age group could easily miss a significant difference when and if one existed.

**Figure 9:**
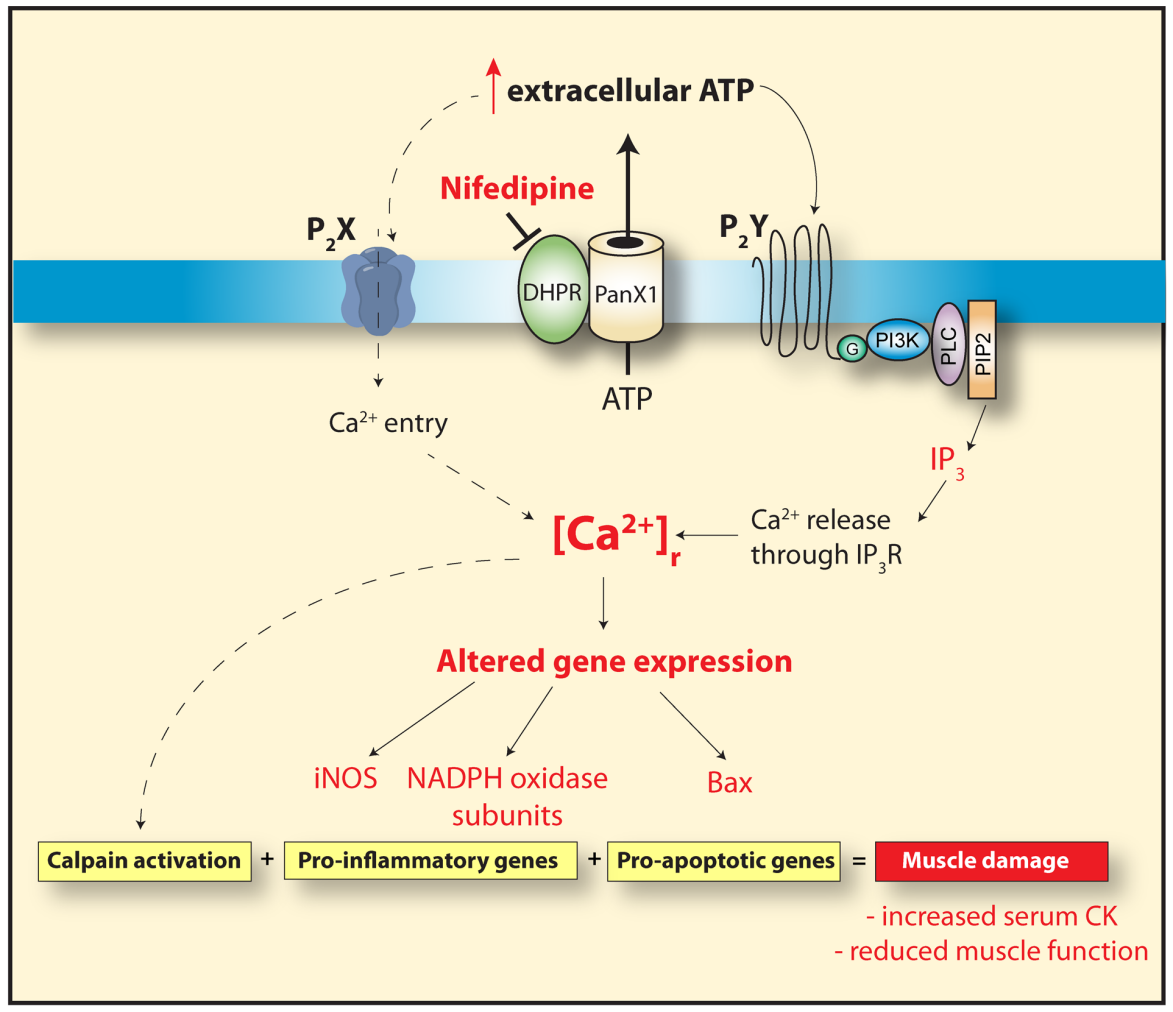
Proposed model for ATP-mediated effects in dystrophic skeletal muscle. In dystrophic skeletal muscle fibers there is an increase in basal ATP release, through Pannexin1 channels that is modulated by the DHPR [12]. Extracellular ATP increases the [Ca^2+^]_r_ in dystrophic muscle fibers through the activation of purinergic receptors (P_2_X, inotropic and P_2_Y, metabotropic). This leads to the expression of pro-apoptotic and pro-inflammatory genes, increasing the muscle damage observed in dystrophic skeletal muscle cells. Nifedipine treatment reduces the basal ATP release and reduces [Ca^2+^]_r,_ resulting in less pro-inflammatory and pro-apoptotic gene expression and subsequently reduces muscle damage as indicated by a decrease in blood CK and an increase in muscle function assessed by the inverted grid-hanging test and the force swimming test.

Reduction of [Ca^2+^]_r_ and in the expression of pro-oxidative/apoptotic proteins, could be beneficial to dystrophic muscles and may explain a reduction in muscle damage that a decrease in serum CK would indicate. Moreover, an increase in the hanging time and normalization of the forced swimming test were observed in nifedipine-treated *mdx* mice, demonstrating an increase in motor function, probably due to reduced muscle damage or increased regeneration. In summary, these results provide further evidence that [Ca^2+^]_r_ is elevated in *mdx* muscles and this can be modulated *in vivo* by nifedipine administration through a reduction in basal ATP release from dystrophic fibers. Moreover, nifedipine treatment reduced pro-oxidative/apoptotic gene expression with the end result being less muscle damage as evidenced by a significant reduction of serum CK and an increased muscle strength in *mdx* mice (Figure 9). Our study used daily intraperitoneally doses of nifedipine (1 mg/Kg) for 1 week as compared to the clinical study, which used it for a longer time period via the oral route of administration. Moreover, our study was carried out in 5–6 week old *mdx* mice (necrosis/regeneration stage) compared to the clinical study that included patients at different stages of the disease (3–27 years old). Longer studies need to be carried out to assess the positive effects and pitfalls of nifedipine treatment studies in *mdx* mice. However, our results strongly suggest that blockers of the ATP signaling pathway should be tested in DMD patients, as they might be promising in palliating the disease and prolonging muscle function.
